# Delays in Presentation Time Under the COVID-19 Epidemic in Patients With Transient Ischemic Attack and Mild Stroke: A Retrospective Study of Three Hospitals in a Japanese Prefecture

**DOI:** 10.3389/fneur.2021.748316

**Published:** 2021-10-27

**Authors:** Koji Tanaka, Shoji Matsumoto, Yusuke Nakazawa, Takeshi Yamada, Kazutaka Sonoda, Sukehisa Nagano, Taketo Hatano, Ryo Yamasaki, Ichiro Nakahara, Noriko Isobe

**Affiliations:** ^1^Department of Neurology, Neurological Institute, Graduate School of Medical Sciences, Kyushu University, Fukuoka, Japan; ^2^Department of Comprehensive Strokology, Fujita Health University School of Medicine, Toyoake, Japan; ^3^Department of Neurosurgery, Kokura Memorial Hospital, Kitakyushu, Japan; ^4^Department of Neurology, Murakami Karindoh Hospital, Fukuoka, Japan; ^5^Department of Neurology, Saiseikai Fukuoka General Hospital, Fukuoka, Japan; ^6^Department of Neurology, Fukuoka City Hospital, Fukuoka, Japan

**Keywords:** ischemic stroke, transient ischemic attack, mild stroke, COVID-19, onset-to-door time

## Abstract

**Background:** Coronavirus Disease 2019 (COVID-19) has spread worldwide with collateral damage and therefore might affect the behavior of stroke patients with mild symptoms seeking medical attention.

**Methods:** Patients with ischemic stroke who were admitted to hospitals within 7 days of onset were retrospectively registered. The clinical characteristics, including onset-to-door time (ODT), of patients with a transient ischemic attack (TIA)/mild stroke (National Institutes of Health Stroke Scale [NIHSS] score of ≤ 3 on admission) or moderate/severe stroke were compared between those admitted from April 2019 to March 2020 (pre-COVID-19 period) and from April to September 2020 (COVID-19 period). Multivariable regression analysis was performed to identify factors associated with the ODT.

**Results:** Of 1,100 patients (732 men, median age, 73 years), 754 were admitted during the pre-COVID-19 period, and 346 were admitted during the COVID-19 period. The number and proportion of patients with TIA/minor stroke were 464 (61.5%) in the pre-COVID-19 period and 216 (62.4%) during the COVID-19 period. Among patients with TIA/mild stroke, the ODT was longer in patients admitted during the COVID-19 period compared with that of the pre-COVID-19 period (median 864 min vs. 508 min, *p* = 0.003). Multivariable analysis revealed the COVID-19 period of admission was associated with longer ODT (standardized partial regression coefficient 0.09, *p* = 0.003) after adjustment for age, sex, route of arrival, NIHSS score on admission, and the presence of hypertension, diabetes mellitus, and wake-up stroke. No significant change in the ODT was seen in patients with moderate/severe stroke.

**Conclusions:** The COVID-19 epidemic might increase the ODT of patients with TIA/mild stroke.

## Introduction

The novel Coronavirus Disease 2019 (COVID-19) has posed a great challenge to the global healthcare system. The reallocation of resources to support the treatment of patients with COVID-19 might have resulted in collateral damage to non-COVID-19-related life-threatening conditions (1). During the pandemic, the number of stroke patients seen in emergency departments has decreased considerably, with a significant reduction in intravenous thrombolysis and endovascular therapy (2–4). Prolongation in onset to hospital arrival time and a significant reduction in individuals arriving at hospitals within 4.5 h were also noted (5–7). In Japan, a state of emergency was declared from April 7 to May 25 2020 because of a massive increase in COVID-19 patients, which had a great impact on the management of acute ischemic stroke patients. Ota et al. (8) reported a rapid decrease in the number of emergent stroke admissions in the Tokyo metropolitan area after the declaration and the trend in restrictions of emergent stroke care systems continued after the declaration was lifted.

Concurrently, several studies showed this decline was more apparent in patients with TIA or mild-to-moderate stroke than for severe stroke (9–11). In a report by Ohara et al. (12), the number of overall stroke patients was decreased, despite increased numbers of stroke patients undergoing thrombectomy or surgery in Kobe City, indicating patients with mild neurological deficits refrained from visiting hospitals during the COVID-19 epidemic. These studies were mainly conducted in the first phase of the COVID-19 pandemic and it remains a rapidly evolving situation. TIA or mild stroke patients are at high risk of subsequent or recurrent ischemic stroke and urgent evaluation and treatment can greatly improve their outcomes (13, 14). Clarifying the impact of the COVID-19 epidemic on the behavior of patients with TIA/mild stroke seeking medical attention may help future public awareness campaigns. Therefore, in this study, we investigated the changes in the clinical characteristics, including onset-to-door time (ODT) in patients with TIA/mild stroke before and during the COVID-19 epidemic using data from three hospitals in Fukuoka Prefecture, Japan.

## Materials and Methods

### Study Design

This was a retrospective study involving three urban hospitals with a stroke care unit located in Fukuoka and Kitakyushu, government-designated cities in Fukuoka Prefecture. Saiseikai Fukuoka General Hospital is a tertiary-level facility and Fukuoka City Hospital is a secondary-level emergency facility for the Fukuoka area including Fukuoka City and surrounding small cities and towns. Kokura Memorial Hospital is a secondary-level emergency facility for the Kitakyushu area. All three hospitals have 24/7 availability of computed tomography, magnetic resonance imaging, and neurologists and/or neurosurgeons to provide stroke care including recanalization therapy. The cohort comprised consecutive patients with acute ischemic stroke admitted within 7 days of onset between April 2019 and September 2020. Trained specialized stroke physicians in each hospital performed emergency evaluations, including imaging, and made decisions about intravenous thrombolysis and endovascular therapy. Patient eligibility for recanalization therapy was determined in accordance with Japanese guidelines (15, 16).

The study period was divided into two parts: pre-COVID-19 (from April 2019 to March 2020) and COVID-19 (from April to September 2020). The beginning of COVID-19 period was defined based on the surge in number of COVID-19 cases in Fukuoka Prefecture and the timing of a declaration of emergency state. On January 16 2020, the first case of COVID-19 was reported in Japan. There was a short delay in the number of cases in Fukuoka Prefecture compared with the Tokyo metropolitan area, but then the number of patients rapidly increased at the end of March 2020. A state of emergency was declared by the Japanese government on April 7 2020. On April 24 2020, the Japan Stroke Society issued a protocol for stroke care during the COVID-19 pandemic (17). According to the protocol, acute stroke treatment was performed under appropriate infection control measures at each institution. The number of new confirmed cases gradually decreased, and the state of emergency was lifted on May 25 2020. However, a second wave occurred in July 2020 (18). Although all three hospitals dedicated their medical resources to treating COVID-19 patients in response to the COVID-19 pandemic, the emergency acceptance of stroke cases was not suspended throughout the study period.

Patient clinical information was recorded in a web-based Research Electronic Data Capture database hosted at Kyushu University Hospital (19). Data were extracted from the hospital discharge record of the Diagnosis Procedure Combination, a mixed-case patient-classification system that includes the principal diagnosis coded according to the International Classification of Diseases and Injuries, 10th revision (ICD-10), which is linked to a hospital finance system (20). We identified patients hospitalized for acute ischemic stroke by using ICD-10 diagnosis codes related to ischemic stroke (I63.0–9) and TIA (G45.0–9) and excluding patients with scheduled admissions. This study was approved by the ethics committee of Kyushu University Hospital (2020-650) and by each facility involved.

### Study Subjects

The following clinical information was systematically collected from medical records: age; sex; history of stroke; the presence of hypertension, diabetes mellitus, dyslipidemia, atrial fibrillation, ischemic heart disease, or congestive heart failure; and maintenance hemodialysis. Route of arrival was categorized as direct walk-in, direct ambulance transport, walk-in with a referral from another medical facility, and transferred from another medical facility. In this study, patients with in-hospital stroke were excluded. Pre-stroke functional status was estimated using the modified Rankin Scale (mRS); pre-stroke disability was defined as an mRS of 3 or more. Because pre-stroke disability potentially affects a patient's behavior related to seeking medical attention and the severity of stroke symptoms on admission, patients with pre-stroke disability were excluded from the analysis. The ODT was defined as the time from onset of stroke or last-known well time to arrival. Wake-up stroke was defined as a stroke occurring during sleep and the last-known well time was bedtime. Acute ischemic lesions were evaluated on admission by computed tomography and/or magnetic resonance imaging including diffusion-weighted imaging. The severity of stroke symptoms was assessed with the National Institutes of Health Stroke Scale (NIHSS) score on admission. A clinical diagnosis of TIA was made if focal neurological symptoms attributable to a vascular etiology lasted for <24 h, without ischemic lesions observed on imaging (21). Minor stroke was defined as transient neurological symptoms or an NIHSS score of ≤ 3 on admission with at least one ischemic lesion (22).

### Statistical Analysis

All statistical analyses were performed with JMP statistical software, version 9.0 (SAS Institute, Cary, NC, USA). Data were expressed as medians and interquartile ranges for continuous variables and counts and percentages for categorical variables.

First, we investigated the trend in the monthly proportions of patients with TIA/mild stroke among overall acute ischemic stroke cases using the Cochran–Armitage trend test. The number of new confirmed COVID-19 cases that were officially announced by Fukuoka Prefecture was also referenced.

Next, for patients with TIA/mild stroke, their clinical characteristics including the ODT were compared between pre-COVID-19 and COVID-19 periods, using the chi-square test, Fisher's exact test, or Wilcoxon rank-sum test. Multivariable regression analysis was performed to investigate factors associated with the ODT, using forced entry and stepwise selection procedures. Age, sex, and the COVID-19 period of admission were forced in; other variables were chosen by stepwise selection with a significance level of α = 0.10 for entry and α = 0.10 for removal, including all variables in the univariate analysis. ODT values were transformed with the Box–Cox transformation to better approximate a normal distribution. The values were back-transformed to facilitate the interpretation of the effect. A similar analysis was performed for patients with diseases other than TIA/mild stroke, representing moderate/severe stroke. A *p*-value of <0.05 was considered statistically significant.

## Results

### Frequencies in TIA/mild Stroke Among Overall Acute Ischemic Stroke Cases During the Study Periods

**Figure 1:**
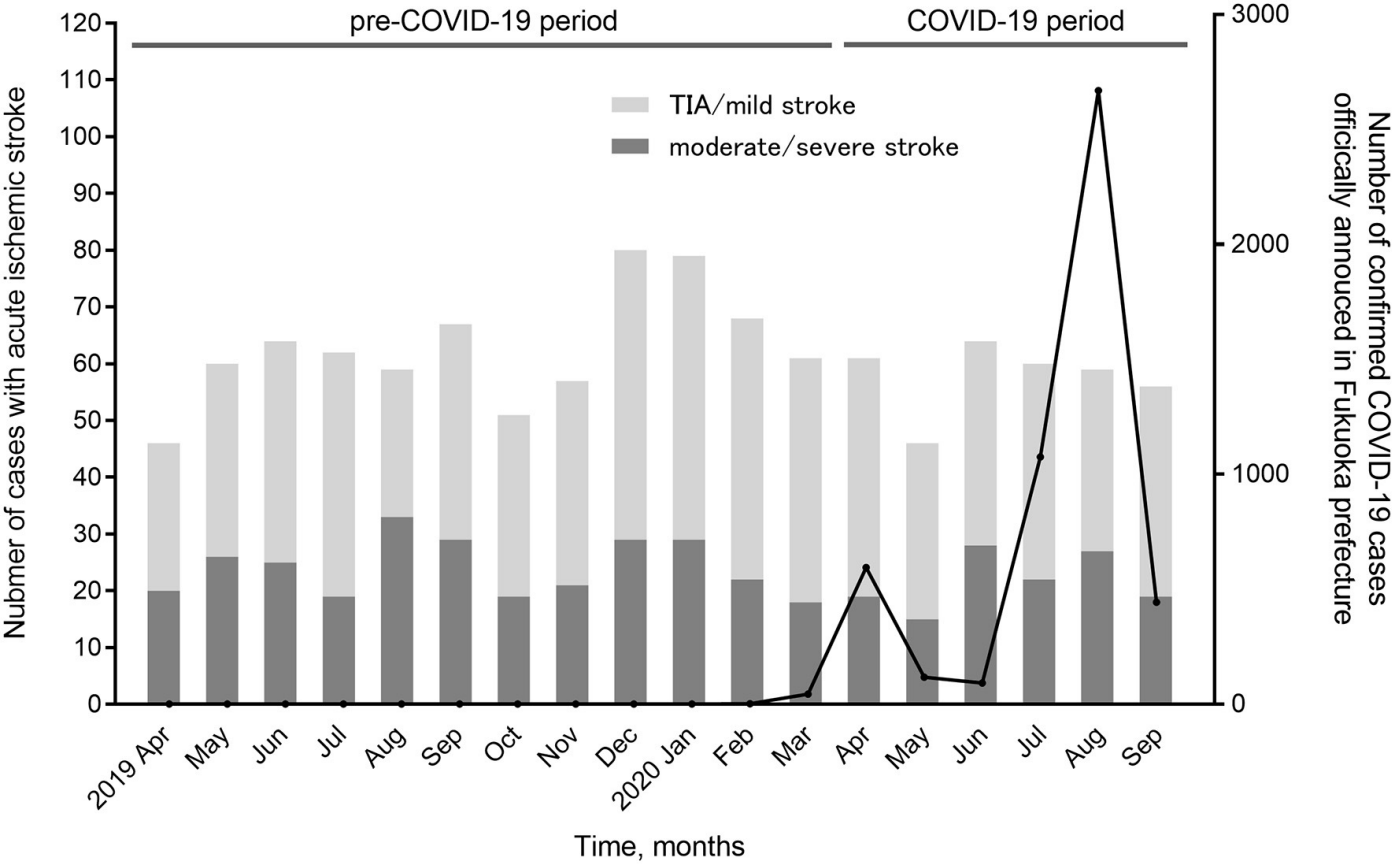
Monthly proportions of patients with transient ischemic attack/mild stroke among overall acute ischemic stroke cases. No significant trend in the proportion of transient ischemic attack (TIA)/mild stroke among overall acute ischemic stroke cases was seen during the study period (*p* = 0.231, Cochran-Armitage trend test).

Overall, 1,571 patients with acute ischemic stroke were admitted during the study period. Among them, 471 patients were excluded because of in-hospital stroke (*n* = 100), asymptomatic or arrived at ≥7 days after onset (*n* = 98), pre-stroke disability (*n* = 184), insufficient clinical information regarding the severity of stroke symptoms (*n* = 18), or an uncertain onset of stroke or last-known well time (*n* = 71). Finally, 1,100 patients (732 men, median age, 73 years) with ischemic stroke (754 admitted during the pre-COVID-19 period and 346 admitted during the COVID-19 period) were included in the analysis. The number and proportion of patients with TIA/minor stroke was 464 (61.5%) in the pre-COVID-19 and 216 (62.4%) in the COVID-19 periods. Figure 1 shows the monthly proportions of patients with TIA/mild stroke among overall acute ischemic stroke cases. There was no significant trend in the proportion of TIA/mild stroke cases during the study period (*p* = 0.231 by Cochran-Armitage trend test). There was one case of stroke with newly confirmed COVID-19 after admission during the study period.

### Changes in Clinical Characteristics During the COVID-19 Epidemic and the Impact of COVID-19 Epidemic on the ODT in Patients With TIA/Mild Stroke

Overall, 680 patients (452 men, median age of 72 years) with TIA/mild stroke (464 admitted during the pre-COVID-19 period and 216 admitted during the COVID-19 period) were included in the analysis. Compared with the pre-COVID-19 period, the route of arrival was different in the COVID-19 period (*p* = 0.034); direct walk-in and direct ambulance transport were decreased (19.0 vs. 24.1% and 31.5 vs. 35.3%, respectively) and walk-in with a referral from another medical facility was increased (39.4% vs. 28.2%). Patients admitted during the COVID-19 period were more likely to have dyslipidemia (53.7 vs. 43.3%, *p* = 0.012) and a longer ODT (median 864 vs. 508 min, *p* = 0.003) compared with those admitted during the pre-COVID-19 period (Table 1). Recanalization therapy was performed for 49 patients (7.2%) with mild stroke during the study period. Intravenous thrombolysis was performed in 34 patients (7.3%) during the pre-COVID-19 period and in 14 patients (6.5%) during the COVID-19 period. Endovascular therapy was performed in two patients (0.4%) in the pre-COVID-19 period. Multivariable analysis revealed that the COVID-19 period of admission was associated with a longer ODT (standardized partial regression coefficient [β] 0.09, *p* = 0.003), after adjustment for age, sex, route of arrival, hypertension, diabetes mellitus, NIHSS score on admission, and wake-up stroke (Table 2), corresponding to 83.0 min delay in original ODT. To exclude the potential impact of seasonal fluctuation on the number of patients with ischemic stroke and the proportion of patients with distinct stroke severity, we performed a sensitivity analysis by extracting 258 patients admitted between April 2019 and September 2020 from the pre-COVID-19 period dataset and compared them with patients in the COVID-19 period (between April 2020 and September 2020). This demonstrated an association between the COVID-19 period of admission and a longer ODT remained significant in the multivariable analysis (β 0.08, *p* = 0.032) (Supplementary Table 1), corresponding to 74.0 min delay in original ODT.

**Table 1:** Characteristics of patients with transient ischemic attack/mild stroke.

**Variables**	**Total**	**Pre-COVID-19 period**	**COVID-19 period**	***p*-value**
	**(*n* = 680)**	**(*n* = 464)**	**(*n* = 216)**	
Sex, male	452 (66.5)	314 (67.7)	138 (63.9)	0.331
Age, years	72 (62–79)	72 (62–80)	71 (60–78)	0.143
History of stroke	132 (19.4)	91 (19.6)	41 (19.0)	0.847
Hypertension	520 (76.5)	350 (75.4)	170 (78.7)	0.349
Dyslipidemia	317 (46.6)	201 (43.3)	116 (53.7)	0.012
Diabetes mellitus	188 (27.6)	128 (27.6)	60 (27.8)	0.959
Atrial fibrillation	92 (13.5)	69 (14.9)	23 (10.6)	0.134
Ischemic heart disease	85 (12.5)	60 (12.9)	25 (11.6)	0.618
Congestive heart failure	26 (3.8)	16 (3.5)	10 (4.6)	0.520
Maintenance hemodialysis	33 (4.9)	26 (5.6)	7 (3.2)	0.250
Route of arrival				0.034
Direct walk-in	153 (22.5)	112 (24.1)	41 (19.0)	
Direct ambulance transport	232 (34.1)	164 (35.3)	68 (31.5)	
Walk-in with a referral from another medical facility	216 (31.8)	131 (28.2)	85 (39.4)	
Transferred from another medical facility	79 (11.6)	57 (12.3)	22 (10.2)	
Wake-up stroke	189 (27.8)	134 (28.9)	55 (25.5)	0.355
Onset-to-door time, min	602 (121–1,461)	508 (105–1,373)	864 (196–1,761)	0.003
Onset-to-door time ≤ 4.5 h	241 (35.4)	180 (38.8)	61 (28.2)	0.007
NIHSS score on admission	1 (0–2)	1 (0–2)	1 (0.25–2)	0.909
Intravenous thrombolysis	48 (7.1)	34 (7.3)	14 (6.5)	0.688
Endovascular therapy	2 (0.3)	2 (0.4)	0 (0)	1.000

*Data are presented as the n (%) or median (interquartile range). NIHSS, National Institutes of Health Stroke Scale*.

**Table 2:** Multivariable regression analyses for the factors associated with onset-to-door time.

**Variable**	**Unstandardized**		**Standardized**	***t* value**	***p-*value**
	**B**	**SE**	**β**		
Intercept	2947.71	131.36		22.44	<0.001
COVID-19 period of admission	67.80	22.68	0.09	2.99	0.003
Wake-up stroke	294.70	24.12	0.37	12.22	<0.001
**Route of arrival**
**Direct walk-in (reference)**
Direct ambulance transport	−194.82	28.68	−0.26	−6.79	<0.001
Walk-in with a referral from another medical facility	207.48	29.05	0.27	7.14	<0.001
Transferred from another medical facility	−27.92	38.16	−0.02	−0.73	0.465
NIHSS on arrival	75.11	19.51	0.11	3.85	<0.001
Diabetes mellitus	60.69	23.76	0.08	2.55	0.011
Hypertension	46.89	25.59	0.06	1.83	0.067
Age	−0.22	1.69	0.00	−0.13	0.895
Sex, male	7.69	22.54	0.01	0.34	0.733
Adjusted R^2^	0.433				

*Onset-to-door time values were transformed by Box-Cox transformation (λ = 0) to better approximate a normal distribution. NIHSS, National Institutes of Health Stroke Scale; B, partial regression coefficient, SE, standard error*.

### Analysis of Patients With Moderate/Severe Stroke in the Pre-COVID-19 and COVID-19 Periods

A similar analysis was performed for patients with moderate/severe stroke. Overall, 420 patients (280 men, median age of 76 years) with moderate/severe stroke (290 admitted during the pre-COVID-19 period and 130 admitted during the COVID-19 period) were included in the analysis. No significant changes in clinical characteristics including the ODT (median 434 vs. 471 min, *p* = 0.952) was observed between the pre-COVID-19 and COVID-19 periods (Supplementary Table 2). Intravenous thrombolysis and endovascular therapy were performed in 82 (28.3%) and in 40 (30.8%) patients during the pre-COVID-19 period and in 78 (26.9%) and 29 (22.3%) patients during the COVID-19 period.

## Discussion

This study clarified the changes in the clinical characteristics of patients with TIA/mild stroke admitted to thrombectomy-capable hospitals during the COVID-19 epidemic. The first major finding of this study was that under the COVID-19 epidemic, a prolongation of ODT occurred despite a comparable case volume and proportion of TIA/mild strokes among overall ischemic strokes during the study period. Similar delays have been described in other settings including acute coronary syndrome, testicular torsion, or pediatric appendicitis (23–27). Previous studies suggest that reluctance to seek medical attention shortly after symptom onset during the COVID-19 pandemic was related to the restrictions in healthcare systems as well as a decreased awareness/shift of attention of the patients, the desire not to overload the emergency healthcare systems, or the fear of in-hospital infection (28). Delays in seeking medical attention after TIA/mild stroke were reported by several studies even before the COVID-19 pandemic (29–32). In addition to the symptoms themselves, the perception and response of patients and people around them, and the referral process contributed to the pre-hospital delay. The independent association between the COVID-19 period of admission and a longer ODT in our study suggests the COVID-19 pandemic is a contributing factor to the delay in presentation time in patients with TIA/mild stroke as a social-psychological behavior modifier.

In contrast to a previous study (5), the ODT was not significantly changed during the COVID-19 epidemic in patients with moderate/severe stroke. However, this was in accordance with the other studies where patients with severe stroke or hemorrhagic stroke were affected less by the COVID-19 pandemic (8–12), indicating that patients and pre-hospital medical staff correctly recognized the need for the emergency care of severe stroke cases. Another possible reason is that emergency acceptance of severe stroke cases was not suspended throughout the study period in these three hospitals, which might be attributable to the relatively low number of COVID-19 cases in Fukuoka Prefecture compared with the Tokyo metropolitan area. Therefore, a massive increase of COVID-19 cases may seriously affect the acute stroke care system.

The second major finding was that the route of arrival changed during the COVID-19 epidemic in patients with TIA/mild stroke: there was an increased frequency of walk-ins with a referral from another facility and a decreased frequency of direct walk-ins and ambulance transport. Although all three hospitals dedicated medical resources to treating COVID-19 patients by implementing infection prevention and control guidelines, patients might have preferred to visit family doctors or their usual or local medical facilities rather than be directly transported to the main hospitals. Our results are consistent with the results of a questionnaire survey reported by Yao et al. (33), where most patients with suspected TIA hesitated to go to a hospital because of the fear of in-hospital infection and complicated procedures, unless a dual attack occurred within a week. Patients with TIA/mild stroke admitted during the COVID-19 period more frequently had dyslipidemia. A similar trend was reported previously (34), although the diagnosis of dyslipidemia was done in the stroke units in this study. The reason for this is unclear but might result from an increase in the frequency of referrals with a detailed history provided by family doctors, or patients without underlying diseases or connections with family doctors refrained from visiting these hospitals directly during the COVID-19 epidemic.

The spread of COVID-19 infection continues globally and therefore, the impact of the COVID-19 pandemic on patients with TIA/mild stoke seeking medical attention may have increased further (35). Continuous research on the impact of the COVID-19 pandemic on patients with TIA/mild stroke seeking medical attention and clinical outcomes is needed. Considering that substantial numbers of patients with mild stroke were eligible for recanalization therapy, it is necessary to continue to increase awareness regarding the need for the urgent evaluation and treatment of TIA/mild stroke.

This study had several limitations. First, this was a retrospective study and was conducted in a small number of facilities with a limited number of patients. A detailed analysis regarding the changes in clinical characteristics within the COVID-19 period (e.g., during and after the state of emergency) could not be performed. Moreover, this study was from one region and might not represent the nationwide situation. Second, outcome data were lacking and the impact of a prolonged ODT on stroke outcomes remains unclear. Previous studies suggest patients with high-risk TIA/minor stroke may have a disadvantage in being treated later especially due to the later administration of secondary preventive treatments. Further study is warranted to elucidate associations between changes in clinical characteristics including the ODT and outcomes in patients with TIA/mild stroke. Third, ABCD^2^ score and some clinical information that might be associated with seeking medical attention [e.g., bystanders, cognitive impairment, initial perception of symptoms, socioeconomic status, or area/infrastructure at residence (36, 37)] were not included in the analysis. Finally, changes in clinical characteristics during the COVID-19 epidemic in patients with pre-stroke disability or in-hospital stroke were not investigated.

## Conclusions

COVID-19 epidemic might increase the ODT in patients with TIA/mild stroke. Public education regarding TIA/minor stroke should be continuously reinforced even during the COVID-19 pandemic.

## Data Availability Statement

The raw data supporting the conclusions of this article will be made available by the authors, without undue reservation.

## Ethics Statement

The studies involving human participants were reviewed and approved by the Ethics Committee of Kyushu University Hospital. Written informed consent for participation was not required for this study in accordance with the national legislation and the institutional requirements.

## Author Contributions

KT, SM, and TY made substantial contributions to the concept and design of the study. KT, YN, KS, SN, and TH contributed to the acquisition, analysis, and interpretation of data. KT, SM, TY, RY, IN, and NI contributed to drafting the text and preparing the figures. All authors contributed to and approved the final manuscript.

## Funding

This research was supported by a grant-in-aid of the International Research Fund for Subsidy of Kyushu University School of Medicine Alumni, Japan.

## Conflict of Interest

The authors declare that the research was conducted in the absence of any commercial or financial relationships that could be construed as a potential conflict of interest.

## Publisher's Note

All claims expressed in this article are solely those of the authors and do not necessarily represent those of their affiliated organizations, or those of the publisher, the editors and the reviewers. Any product that may be evaluated in this article, or claim that may be made by its manufacturer, is not guaranteed or endorsed by the publisher.

## References

[1] Feral-Pierssens AL Claret PG Chouihed T. Collateral damage of the COVID-19 outbreak: expression of concern. Eur J Emerg Med. (2020) 27:233–34. 10.1097/MEJ.0000000000000717 32345850 PMC7202126

[2] Kiss P Carcel C Hockham C Peters SAE. The impact of the COVID-19 pandemic on the care and management of patients with acute cardiovascular disease: a systematic review. Eur Heart J Qual Care Clin Outcomes. (2021) 7:18–27. 10.1093/ehjqcco/qcaa084 33151274 PMC7665454

[3] Nogueira RG Qureshi MM Abdalkader M Martins SO Yamagami H Qiu Z. Global impact of COVID-19 on stroke care and IV thrombolysis. Neurology. (2021) 96:e2824–38. 10.1212/WNL.0000000000011885 33766997 PMC8205458

[4] Nogueira RG Abdalkader M Qureshi MM Frankel MR Mansour OY Yamagami H. Global impact of COVID-19 on stroke care. Int J Stroke. (2021) 16:573–84. 10.1177/1747493021991652 33459583 PMC8010375

[5] Schirmer CM Ringer AJ Arthur AS Binning MJ Fox WC James RF. Delayed presentation of acute ischemic strokes during the COVID-19 crisis. J Neurointerv Surg. (2020) 12:639–42. 10.1136/neurintsurg-2020-016299 32467244

[6] Teo KC Leung WCY Wong YK Liu RKC Chan AHY Choi OMY. Delays in stroke onset to hospital arrival time during COVID-19. Stroke. (2020) 51:2228–31. 10.1161/STROKEAHA.120.030105 32432998 PMC7258759

[7] Nagamine M Chow DS Chang PD Boden-Albala B Yu W Soun JE. Impact of COVID-19 on acute stroke presentation at a comprehensive stroke center. Front Neurol. (2020) 11:850. 10.3389/fneur.2020.00850 32922355 PMC7456804

[8] Ota T Shiokawa Y Hirano T. Impact of COVID-19 on stroke admissions and the medical care system in the tokyo metropolitan area. Front Neurol. (2020) 11:601652. 10.3389/fneur.2020.601652 33424751 PMC7793774

[9] Diegoli H Magalhães PSC Martins SCO Moro CHC França PHC Safanelli J. Decrease in hospital admissions for transient ischemic attack, mild, and moderate stroke during the COVID-19 era. Stroke. (2020) 51:2315–21. 10.1161/STROKEAHA.120.030481 32530738 PMC7302100

[10] Butt JH Fosbøl EL Østergaard L Yafasova A Andersson C Schou M. Effect of COVID-19 on first-time acute stroke and transient ischemic attack admission rates and prognosis in denmark: a nationwide cohort study. Circulation. (2020) 142:1227–9. 10.1161/CIRCULATIONAHA.120.050173 32755320 PMC7497886

[11] Uphaus T Gröschel S Hayani E Hahn M Steffen F Gröschel K. Stroke care within the COVID-19 pandemic-increasing awareness of transient and mild stroke symptoms needed. Front Neurol. (2020) 11:581394. 10.3389/fneur.2020.581394 33154735 PMC7586312

[12] Ohara N Imamura H Adachi H Hara Y Hosoda K Kimura H. Stroke systems of care during the COVID-19 epidemic in Kobe city. J Stroke Cerebrovasc Dis. (2020) 29:105343. 10.1016/j.jstrokecerebrovasdis.2020.105343 33039766 PMC7526598

[13] Lavallée PC Meseguer E Abboud H Cabrejo L Olivot JM Simon O. A transient ischaemic attack clinic with round-the-clock access (SOS-TIA): feasibility and effects. Lancet Neurol. (2007) 6:953–60. 10.1016/S1474-4422(07)70248-X 17928270

[14] Rothwell PM Giles MF Chandratheva A Marquardt L Geraghty O Redgrave JN. Effect of urgent treatment of transient ischaemic attack and minor stroke on early recurrent stroke (EXPRESS study): a prospective population-based sequential comparison. Lancet. (2007) 370:1432–42. 10.1016/S0140-6736(07)61448-2 17928046

[15] Toyoda K Koga M Iguchi Y Itabashi R Inoue M Okada Y. Guidelines for intravenous thrombolysis (recombinant tissue-type plasminogen activator), the third edition, March 2019: a guideline from the Japan stroke society. Neurol Med Chir. (2019) 59:449–91. 10.2176/nmc.st.2019-0177 31801934 PMC6923159

[16] Yamagami H Hayakawa M Inoue M Iihara K Ogasawara K Toyoda K. Guidelines for mechanical thrombectomy in Japan, the fourth edition, March 2020: a guideline from the Japan stroke society, the Japan neurosurgical society, and the Japanese society for neuroendovascular therapy. Neurol Med Chir. (2021) 61:163–92. 10.2176/nmc.nmc.st.2020-0357 33583863 PMC7966209

[17] Japanese Stroke Society PCS Working Group. Protocol for stroke management during COVID-19 pandemic: protected code stroke, Japan Stroke Society edition (JSS-PCS). Jpn J Stroke. (2020) 42:315–43. 10.3995/jstroke.10828

[18] Saito S Asai Y Matsunaga N Hayakawa K Terada M Ohtsu H. First and second COVID-19 waves in Japan: a comparison of disease severity and characteristics. J Infect. (2021) 82:84–123. 10.1016/j.jinf.2020.10.033 33152376 PMC7605825

[19] Harris PA Taylor R Thielke R Payne J Gonzalez N Conde JG. Research electronic data capture (REDCap)–a metadata-driven methodology and workflow process for providing translational research informatics support. J Biomed Inform. (2009) 42:377–81. 10.1016/j.jbi.2008.08.010 18929686 PMC2700030

[20] Yasunaga H Ide H Imamura T Ohe K. Impact of the Japanese diagnosis procedure combination-based payment system on cardiovascular medicine-related costs. Int Heart J. (2005) 46:855–66. 10.1536/ihj.46.855 16272776

[21] Easton JD Saver JL Albers GW Alberts MJ Chaturvedi S Feldmann E. Definition and evaluation of transient ischemic attack: a scientific statement for healthcare professionals from the American heart association/american stroke association stroke council; council on cardiovascular surgery and anesthesia; council on cardiovascular radiology and intervention; council on cardiovascular nursing; and the interdisciplinary council on peripheral vascular disease. the American academy of neurology affirms the value of this statement as an educational tool for neurologists. Stroke. (2009) 40:2276–93. 10.1161/STROKEAHA.108.192218 19423857

[22] Fischer U Baumgartner A Arnold M Nedeltchev K Gralla J De Marchis GM. What is a minor stroke? Stroke. (2010) 41:661–6. 10.1161/STROKEAHA.109.572883 20185781

[23] Holzman SA Ahn JJ Baker Z Chuang KW Copp HL Davidson J. A multicenter study of acute testicular torsion in the time of COVID-19. J Pediatr Urol. (2021) 17:e1 478–e6 478. 10.1016/j.jpurol.2021.03.013 33832873 PMC7977032

[24] Fisher JC Tomita SS Ginsburg HB Gordon A Walker D Kuenzler KA. Increase in pediatric perforated appendicitis in the New York city metropolitan region at the epicenter of the COVID-19 outbreak. Ann Surg. (2021) 273:410–5. 10.1097/SLA.0000000000004426 32976285 PMC7869969

[25] Roffi M Guagliumi G Ibanez B. The obstacle course of reperfusion for ST-segment-elevation myocardial infarction in the COVID-19 pandemic. Circulation. (2020) 141:1951–3. 10.1161/CIRCULATIONAHA.120.047523 32315205 PMC7294591

[26] Popovic B Varlot J Metzdorf PA Jeulin H Goehringer F Camenzind E. Changes in characteristics and management among patients with ST-elevation myocardial infarction due to COVID-19 infection. Catheter Cardiovasc Interv. (2021) 97:E319–26. 10.1002/ccd.29114 32667726 PMC7405489

[27] Toner L Koshy AN Hamilton GW Clark D Farouque O Yudi MB. Acute coronary syndromes undergoing percutaneous coronary intervention in the COVID-19 era: comparable case volumes but delayed symptom onset to hospital presentation. Eur Heart J Qual Care Clin Outcomes. (2020) 6:225–6. 10.1093/ehjqcco/qcaa038 32379888 PMC7239230

[28] Wong LE Hawkins JE Langness S Murrell KL Iris P Sammann A. Where are all the patients? addressing Covid-19 fear to encourage sick patients to seek emergency care. NEJM Cat Innov Care Delivery. (2020). 10.1056/CAT.20.0193

[29] Sprigg N Machili C Otter ME Wilson A Robinson TG. A systematic review of delays in seeking medical attention after transient ischaemic attack. J Neurol Neurosurg Psychiatry. (2009) 80:871–5. 10.1136/jnnp.2008.167924 19273474

[30] Mc Sharry J Baxter A Wallace LM Kenton A Turner A French DP. Delay in seeking medical help following transient ischemic attack (TIA) or “mini-stroke”: a qualitative study. PLoS ONE. (2014) 9:e104434. 10.1371/journal.pone.0104434 25137185 PMC4138063

[31] Dolmans LS Hoes AW Bartelink MEL Koenen NCT Kappelle LJ Rutten FH. Patient delay in TIA: a systematic review. J Neurol. (2019) 266:1051–8. 10.1007/s00415-018-8977-6 30027321 PMC6469675

[32] Dolmans LS Kappelle LJ Bartelink ME Hoes AW Rutten FH. Delay in patients suspected of transient ischaemic attack: a cross-sectional study. BMJ Open. (2019) 9:e027161. 10.1136/bmjopen-2018-027161 30819716 PMC6398704

[33] Yao S Lin B Liu Y Luo Y Xu Q Huang J. Impact of Covid-19 on the behavior of community residents with suspected transient ischemic attack. Front Neurol. (2020) 11:590406. 10.3389/fneur.2020.590406 33178128 PMC7596267

[34] Jasne AS Chojecka P Maran I Mageid R Eldokmak M Zhang Q. Stroke code presentations, interventions, and outcomes before and during the COVID-19 pandemic. Stroke. (2020) 51:2664–73. 10.1161/STR.0000000000000347 32755347 PMC7446978

[35] Karako K Song P Chen Y Tang W Kokudo N. Overview of the characteristics of and responses to the three waves of COVID-19 in Japan during 2020-2021. Biosci Trends. (2021) 15:1–8. 10.5582/bst.2021.01019 33518668

[36] Jin H Zhu S Wei JW Wang J Liu M Wu Y. Factors associated with prehospital delays in the presentation of acute stroke in urban China. Stroke. (2012) 43:362–70. 10.1161/STROKEAHA.111.623512 22246693

[37] Seo AR Song H Lee WJ Park KN Moon J Kim D. Factors associated with delay of emergency medical services activation in patients with acute stroke. J Stroke Cerebrovasc Dis. (2021) 30:105426. 10.1016/j.jstrokecerebrovasdis.2020.105426 33161352

