# Supplementary material for: Delays in Presentation Time Under the COVID-19 Epidemic in Patients With Transient Ischemic Attack and Mild Stroke: A Retrospective Study of Three Hospitals in a Japanese Prefecture
Source: Front Neurol. 2021 Oct 27;12:748316. doi: 10.3389/fneur.2021.748316 (PMC8578818; doi:10.3389/fneur.2021.748316)
Supplement: Supplementary file 1 [file Table_1.docx]

**Supplementary Table 1.** Multivariable regression analyses for the factors associated with onset-to-door time.

| Variable | Unstandardized | |  | Standardized | *t* value | *p-*value |
| --- | --- | --- | --- | --- | --- | --- |
|  | B | SE |  | β |  |  |
| Intercept | 3242.62 | 195.11 |  |  | 16.62 | <0.001 |
| COVID-19 period of admission | 64.35 | 29.93 |  | 0.08 | 2.15 | 0.032 |
| Wake-up stroke | 310.68 | 33.44 |  | 0.36 | 9.29 | <0.001 |
| Route of arrival |  |  |  |  |  |  |
| Direct walk-in (reference) |  |  |  |  |  |  |
| Direct ambulance transport | −216.15 | 40.14 |  | −0.26 | −5.39 | <0.001 |
| Walk-in with a referral from another medical facility | 181.14 | 40.76 |  | 0.22 | 4.44 | <0.001 |
| Transferred from another medical facility | −36.34 | 52.01 |  | −0.03 | −0.70 | 0.485 |
| NIHSS on arrival | 85.15 | 27.53 |  | 0.12 | 3.09 | 0.002 |
| Ischemic heart disease | 131.46 | 45.19 |  | 0.11 | 2.91 | 0.004 |
| Diabetes mellitus | 76.06 | 34.39 |  | 0.09 | 2.21 | 0.028 |
| Hypertension | 64.85 | 35.75 |  | 0.07 | 1.81 | 0.070 |
| Age | −2.10 | 2.40 |  | −0.03 | −0.87 | 0.383 |
| Sex, male | −31.13 | 31.71 |  | −0.04 | −0.98 | 0.327 |
| Adjusted R^2^ | 0.406 |  |  |  |  |  |

Onset-to-door time values were transformed by Box-Cox transformation (λ = 0) to better approximate a normal distribution.

Abbreviations: NIHSS, National Institutes of Health Stroke Scale; B, partial regression coefficient; SE, standard error.

**Supplementary Table 2.** Characteristics of patients with moderate/severe stroke.

| Variables | Total  (*n* = 420) | Pre-COVID-19 period  (*n* = 290) | COVID-19 period  (*n* = 130) | *p*-value |
| --- | --- | --- | --- | --- |
| Sex, male | 280 (66.7) | 193 (66.6) | 87 (66.9) | 0.941 |
| Age, years | 76 (67–82) | 77 (69–83) | 75 (66–82) | 0.246 |
| History of stroke | 92 (21.9) | 63 (21.7) | 29 (22.3) | 0.894 |
| Hypertension | 294 (70.0) | 203 (70.0) | 91 (70.0) | 1.000 |
| Dyslipidemia | 170 (39.0) | 113 (39.0) | 57 (43.8) | 0.346 |
| Diabetes mellitus | 114 (27.1) | 78 (26.9) | 36 (27.7) | 0.865 |
| Atrial fibrillation | 121 (28.8) | 89 (30.7) | 32 (24.6) | 0.204 |
| Ischemic heart disease | 50 (11.9) | 33 (11.4) | 17 (13.1) | 0.619 |
| Congestive heart failure | 37 (8.8) | 26 (9.0) | 11 (8.5) | 0.866 |
| Maintenance hemodialysis | 16 (3.8) | 8 (2.8) | 8 (6.2) | 0.103 |
| Route of arrival |  |  |  | 0.068 |
| Direct walk-in | 31 (7.4) | 21 (7.2) | 10 (7.7) |  |
| Direct ambulance transport | 264 (62.9) | 188 (64.8) | 76 (58.5) |  |
| Walk-in with a referral from another medical facility | 54 (12.9) | 29 (10.0) | 25 (19.2) |  |
| Transferred from another medical facility | 71 (16.9) | 52 (17.9) | 19 (14.6) |  |
| Wake-up stroke | 145 (34.5) | 106 (36.6) | 39 (30.0) | 0.192 |
| Onset-to-door time | 441 (88–964) | 434 (96–967) | 471 (74–982) | 0.952 |
| Onset-to-door time ≤4.5 h | 179 (42.6) | 120 (41.4) | 59 (45.4) | 0.443 |
| NIHSS score on admission | 7 (5–15) | 7 (5–15) | 6 (4–14) | 0.909 |
| Intravenous thrombolysis | 122 (29.0) | 82 (28.3) | 40 (30.8) | 0.603 |
| Endovascular therapy | 107 (25.5) | 78 (26.9) | 29 (22.3) | 0.318 |

Data are presented as the n (%) or median (interquartile range).

Abbreviation: NIHSS, National Institutes of Health Stroke Scale.
